# Cellular Auxin Transport in Algae

**DOI:** 10.3390/plants3010058

**Published:** 2014-01-27

**Authors:** Suyun Zhang, Bert van Duijn

**Affiliations:** 1 Plant Biodynamics Laboratory, Biology Institute Leiden, Leiden University, Sylviusweg 72, Leiden 2333, BE, The Netherlands; E-Mail: s.zhang@biology.leidenuniv.nl; 2 Fytagoras, Sylviusweg 72, Leiden 2333, BE, The Netherlands

**Keywords:** auxin, algae, polar auxin transport, *Chara*

## Abstract

The phytohormone auxin is one of the main directors of plant growth and development. In higher plants, auxin is generated in apical plant parts and transported from cell-to-cell in a polar fashion. Auxin is present in all plant phyla, and the existence of polar auxin transport (PAT) is well established in land plants. Algae are a group of relatively simple, autotrophic, photosynthetic organisms that share many features with land plants. In particular, Charophyceae (a taxon of green algae) are closest ancestors of land plants. In the study of auxin function, transport and its evolution, the algae form an interesting research target. Recently, proof for polar auxin transport in *Chara species* was published and auxin related research in algae gained more attention. In this review we discuss auxin transport in algae with respect to land plants and suggest directions for future studies.

## 1. Introduction

Auxins are a class of plant hormones with morphogen-like characteristics that regulate the rate of cell division, cell elongation and expansion, ethylene biosynthesis, apical dominance, organ differentiation and some other essential processes in plant growth [1,2].

In the late 19th century, Charles Darwin investigated “the power of movement in plants” [3]. However, it took nearly a century before, in 1926, Fritz Went opened a new field of study by successfully isolating “the plant growth substance” auxin [4,5]. Later he introduced the avena curvature test enabling many experimenters to be active in auxin research [6,7]. Now it is known that auxin is locally synthesized in (land) plants, mainly in shoot tissues, from where it is distributed unevenly throughout the whole plant [5]. Concentration differences of auxin can cause different responses in plant development. During the early 20th century, the traditional “cut-off and stuck-on” tests and later the use of ^14^C-indoleacetic acid showed that auxin is distributed through the plant by energy-requiring polar transport, with three important characteristics: chemically highly specific, oxygen-requiring and pH dependent [8].

The most abundant naturally occurring auxin is indole-3-acetic acid (IAA) which shows characteristic “polar transport” throughout the whole plant [9,10,11,12]. Mid 1970s, based on the known properties of auxin movement (saturable, energy- and protein synthesis-dependent and unidirectional), the “chemiosmotic hypothesis” was formulated as a classic model to explain the mechanism of polar auxin transport (PAT) [10,13]. According to this model, IAA can easily enter the cell cytoplasm in an undissociated lipophilic form (IAAH) when in a slightly acidic extracellular environment (pH 5.5). While in the cytosol, with a neutral pH of about 7, most of the IAA will be dissociated into anions (IAA^−^) and, therefore, trapped inside the cell. To aid the exit of IAA^−^ active auxin anion efflux carriers were proposed, and the asymmetric distribution of such carriers was postulated to attribute to the directionality of auxin transport [14]. This hypothesis later has been proven to be suitable to describe in general auxin transport in land plants [13].

Although auxins are shown to be present in early-diverged plant lineages such as mosses, liverworts and algae [15,16], research on auxin transport and signaling remains mainly focused on seed plants, predominantly in the model plant *Arabidopsis thaliana* [17]. However, algae share many specialized characteristics with the land plants while evolutionally earlier and of simpler structures. Hence, they may provide unique features that may help to unravel (new) auxin working mechanisms, auxin transport characteristics and functions. In this paper, we look into auxin as a plant hormone in algae, and specially focus on auxin transport.

## 2. The Roles of Auxin in Algae

Algae belong to a very large and diverse group of simple, typically autotrophic organisms. Increasingly data appears that all land plants (embryophytes) diverged from ancestral *Charophycean* algae, a class of green algae, about 400–500 million years ago, Table 1 [18,19]. Table 1 also shows that the red algae and brown algae are more distant from the streptophytes. In this respect, green algae especially members in the class of Charophyceae are promising candidates to study evolutionary aspects, (new) functionalities and cellular physiology of auxin.

Bioassays, high-performance liquid chromatography, mass spectrometry and some other physicochemical analysis, together with other circumstantial evidences, proof the existence of auxin or auxin-like compounds in many species of algae [17,20,21]. The measured concentrations in these studies are highly variable. The presence and action of auxins have been shown both in unicellular and multi-cellular algae [9,17,22,23,24]. For example, in red algae (e.g., *Grateloupia dichotoma*, *Gracilaria vermiculophylla*, *Agardhiella subulata*) and green algae (e.g., *Chlorella pyrenoidosa*, *Micrasterias thomasiana*) auxin stimulates cell division and cell enlargement [25,26,27,28,29,30] and affects the development and growth of rhizoids as well [31,32,33]. Although the full-value of plant hormone systems in algae is still under debate, the aforementioned studies about auxins on algal growth and development indicate that its functions likely correspond to its activity in higher land plants [20,34,35]. As studies so far concentrated on land plant corresponding functions other, new and unexpected, roles for auxins in algae may have been overlooked and remain to be discovered.

**Table 1:** Partial classification of plants and brown algae.

Kingdom	Division	Class	Order
Plantae	Chlorophyta (green algae)		
	Streptophyta	**Charophyceae** (green algae)	Zygnematales, Coleochaetales, Charales, Desmidiales, Klebsormidiales, Mesostigmatales
		**Embryophyceae** (land plants:mosses lato sensu, lycophytes, ferns and horsetails, seed plants)	
	Rhodophyta (red algae)		
Chromalveolata	Heterokontophyta	Phaeophyceae (brown algae)	

## 3. Transport of Auxin in Algae

Auxin acts both as hormone and morphogen. The role of auxin in apical dominance can be regarded as a classical example of hormonal integration based on hormone distribution in land plants. Similar phenomena have also been described in various seaweeds, suggesting that in algae similar auxin distribution systems and carriers supporting PAT may be present [34,36].

The events in auxin signaling as established in seed plants do not only involve the sensing of auxins at the level of the target cells and their responses but also auxin biosynthesis and metabolism, intracellular compartmentalization, and directional transport through cells facilitated by specific transporters [37,38].

Auxin transport in seed plants is characterized by its polarity, directionality, distance, and transporting cells [17,39]. Polar auxin transport (PAT) is facilitated by influx carriers (AUX1/LAX proteins) and efflux carriers (PIN proteins), together with some other transport proteins (ABCB/PGP transporter family) [11,13]. The asymmetric distribution of efflux-carriers, mainly related to the plasma-membrane-localized PINs, aids to the gradient of auxin concentration through the whole plant, while the differences of auxin concentration in turn regulate the number and location of efflux-carriers [14,40,41,42]. The endoplasmic reticulum (ER)-localized PIN proteins (like PIN5, PIN8) are thought to charge the intracellular compartmentalization of auxin and homeostasis, in cooperation with members of the recently-found auxin carrier family PIN-LIKEs (PILS) [43,44,45].

To understand this whole system better, splitting it into several parts and searching them back in the evolutionarily earlier organisms of green or brown algae lineages will be helpful.

In view of some of the apparent morphological similarities between algae and land plants one might ask if the ability of auxin polar transport is required for such differential development? Studies in the large coenocytic (multi-cellular structures without cross walls) green alga *Caulerpa paspaloides* show different results. Although these algae show characteristics like a leaf-like assimilator which grows up, rhizoid clusters that grow down, and a rhizome that grows horizontally, auxin displays an uniform and non-polar distribution, which might be caused by diffusion and cytoplasmic streaming [22,46,47]. This suggests that auxin polar transport and auxin gradients do not participate in, at least, the later development and maintenance of these three different organs. In multi-cellular algae the existence of PAT has also been investigated. In *Chara*, a branched, multi-cellular green alga, a specific carrier system is suggested to mediate the transmembrane auxin transport [46]. This carrier lacked the inhibition by 1-*N*-Naphthylphthalamic acid (NPA, a phytotropin) which is typical for inhibiting efflux carriers in land plants [46]. In the same study, such specific auxin carriers were not found in the simple unicellular green alga *Chlorella vulgaris* [46]. On the other hand, Klämbt and coworkers reported that in growing rhizoids of *Chara*, auxin efflux showed NPA-sensitive activation [32]. These contradictory results are thought to be due to some additional effects of NPA, unrelated to its ability to increase intracellular IAA levels by blocking IAA efflux [17]. Recent experiments, showing NPA sensitive polar transport of radioactive labeled auxin, provide more direct proof of the presence of PAT in *Chara* and suggest the presence of specific auxin efflux carriers as in land plants [48]. In addition, PAT has been shown to exist in brown algae species like *Fucus distichus* and *Ectocarpus siliculosus* [31,49].

Comparing studies in unicellular (micro or coenocytic) green algae with multi-cellular green algae (*Chara*), Dibb-Fuller and Morris proposed that “the appearance of specific auxin carrier systems in the *Chlorophyta* may have been functionally associated with the evolution of multi-cellularity, rather than with the evolution of a plant body which is characterized by distinctly different morphological regions” [46]. This conclusion seems in accordance with the early hypothesis raised by others, that after the development of multicellular organisms, simple diffusion of IAA would not be efficient enough, the movement across cell membranes is required for polar transport of IAA [8,47,50]. Although the relatively lipid-soluble IAA could account for very slow diffusive transport through membranes, for more effective longer distance transport and gradients in tissue, membrane bound auxin transporters and channels are required.

Since PAT in seed plants is largely dependent on the asymmetric distribution of PIN proteins, the PIN proteins seem a key in the investigation of PAT mechanisms. Though the PILS carrier family is quite conserved throughout the evolution of plants and can be found from unicellular algae to highly developed seed plants, the PIN exporter families at the plasma membrane are comparatively young in evolutionarily perspective [37,51]. The plasma membrane-localized PIN proteins are thought to exist only in land plants, while the endoplasmic reticulum (ER)-localized PIN proteins (like PIN5, PIN8 in *Arabidopsis*) are evolutionarily older, and can be traced back to an origin in Streptophyta algae [51].

By using a basic local alignment search tool (BLAST), partial PIN protein sequences can be identified in several species of green algae like *Spirogyra*, *Penium*, and the evolutionarily even earlier lineage *Klebsormidiophyceae* [51,52]. However, the above results are based on the expressed sequence tag (EST) database, since the nuclear genomes sequences database is barely described in the multicellular green algae of the Streptophyta group. The scant annotated genomes are almost all from the unicellar algae of the Chlorophyta group, and there is not yet evidence of the existence of PIN protein sequences [37,51]. With the growing information on nuclear genomes of multicellular green algae groups, a clearer picture is soon expected on the presence of PIN proteins.

Although evidence for functional PIN proteins supporting PAT in algae is (still) scarce this does not rule out the (functional) existence of PAT in algae. In the brown algae *Ectocarpus siliculosus* PAT has been shown while this species lacks any PIN homolog [53]. The possibility of other types of auxin (polar) transporter mechanisms rather than PIN proteins cannot be excluded. Besides, the presence of plasmodesmata or other active (e.g., vesicle based) transport systems may exist. Researches have shown that there exists a special plasma membrane invagination-structure named charasomes in Chara species, but not in Nitella species. Interestingly, PAT also shows up in *Chara corallina*, but not in Nitella [48,54,55,56,57]. Charasomes are thought to be closely related to the ability of endocytosis in Chara and the origin of vesicles [54]. Although even if it is possible for auxin to be transported in vesicles, the driving force of these vesicles in these algae species remains a mystery: the block of cytostreaming by cytochalasin H could not stop the auxin transport in Chara while the normal microtubule system seems not fast enough to reach the observed speed of auxin transport. This suggests the existence of an unknown amplification mechanism [48,58]. Taking everything into consideration, it is still a challenge to identify possible PIN protein independent polar auxin transport mechanisms that may have emerged in the early period of evolution, and may or may not have been eliminated during the evolution of land plants. Algae form an attractive model to investigate these potentially early systems and learn more about the basic properties of auxin transport and auxin function.

**Figure 1:**
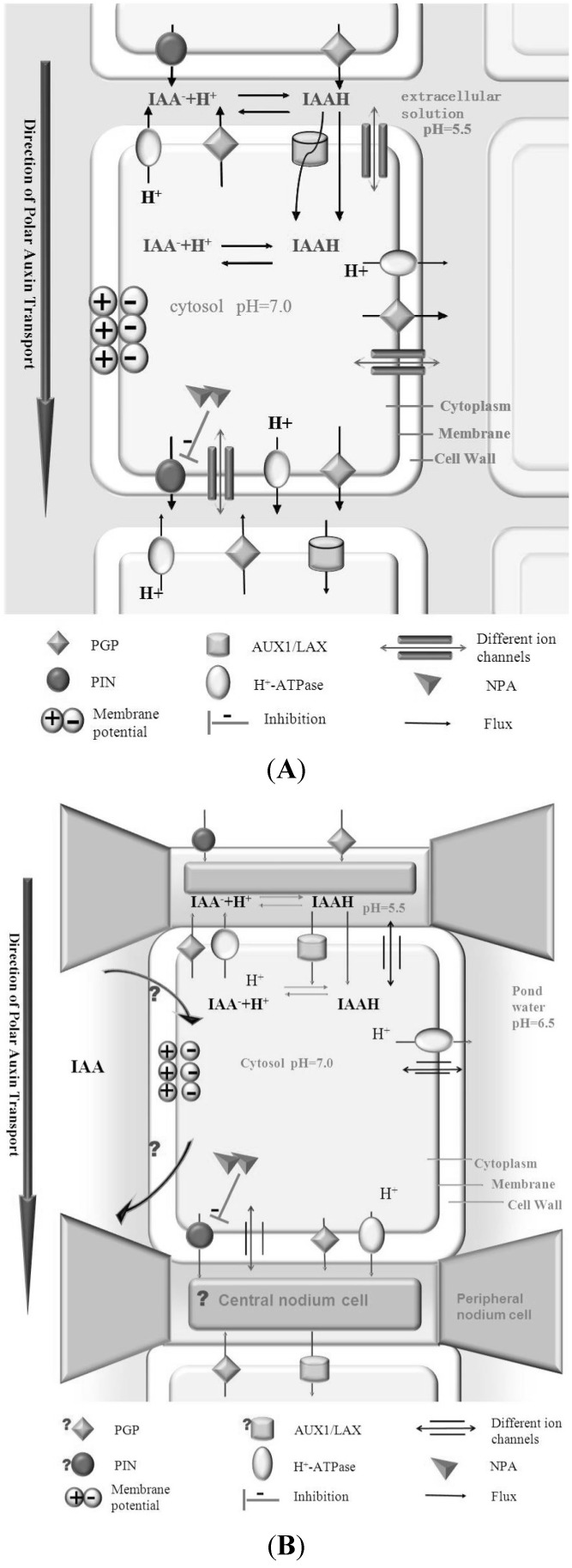
(**A**) Auxin transport in seed plants; (**B**) The hypothetical auxin transport system in *Chara*.

At present, our knowledge on the role of auxin and its transport in algae is still very limited. However, we are still able to draw some conclusions based on the available studies. With regard to (polar) auxin transport, Figure 1 illustrates a summary and comparison between land plants and multi-cellular algae, like *Chara corallina.* In both models PAT is present based on auxin influx carriers and NPA sensitive efflux carriers, and a chemiosmotic mechanism. Although the presence of NPA sensitive PAT in *Chara* in combination with our understanding of PAT in land plants justifies this hypothetical model, it must be emphasized that the hypothetical presence of these specific transport proteins in algae is only based on indirect evidence. In addition, the membrane potential difference across the plasmalemma should also be taken into consideration as this plays a significant role in the chemiosmotic mechanism, and complicates the auxin transport model [59,60,61]. Compared with our knowledge the model system of seed plants—Arabidopsis, studies on algae are still facing many challenges, especially with regard to the still insufficient genetic background database and possibilities for application of molecular biology tools.

## 4. Algae as Model System

Research in the field of auxin and algae were mainly on the brown algae when related to the function of auxin, while the green algae were more favorable in studies related to signaling and transport. In view of modern phylogenetics, the division Charophyta contains the closest living relatives to the land plants. In this division, the order Charales has been widely accepted as a sister-relationship to the land plants over the past century, which was based also on the morphology since the Charales develop the most complicated, land-plant-like body structures as compared to all the other orders [62]. Now the phenomenon of PAT is observed in *Chara*, a genera of Charales, showing that, after the discovery of PAT in moss species (*Physcomitrella patens*) [49,63], these green algae species are so far the earliest life forms showing PAT in the non-land plants [46]. Recent research data show that Zynematales and Coleochaetales are more close to the land plants than Charales [64,65,66] and it has been proposed that the grade of organismal complexity of Charales may have evolved independently. It remains to be discovered whether the Charales share a similar auxin polar transport mechanism with the land plants or have evolved a different mechanism of PAT. Therefore, from an evolutionary point of view, auxin related research aiming at early mechanisms and functions should be focused on these organisms. Within the order of *Charales*, species from the genera *Chara* have shown a great potential for the future studies in auxin signaling and transport. Species of *Chara* have the giant internodal cells that provide an ideal and unique model for polar auxin transport research at the single cell level while they do show the ability of polar transport of auxin. To some extent, these cells are superior to the model system *Arabidopsis thaliana*, such as for cell biological and cell physiology studies of auxin transport and signaling at the single cell level as advanced microscopy, electrophysiology, and transport studies that can be applied to the intact cell under natural conditions.

Using algae in auxin research requires some precautions and approaches that are less relevant in studies on land plant. For instance, the use of non-sterile algae materials could lead to false conclusions as bacteria or secondary algae may produce auxin-like substances or break down endogenous algal auxin. Some special cautions are also necessary in choice and conditioning of cells for auxin transport studies, especially since the function of auxin in algae remains unclear. In our recent experience, we noticed that in some situations the *Chara* internodes may not show the PAT phenomenon (data not show). This could be an example of auxin feedback regulation on the efflux carriers. The reasons and details need a further study. Also, in higher plants, such effects have been reported. Decapitation of growing shoots can result in the loss of polar auxin transport in segments from internodes subtending the apex [67].

Besides the possibility that “apical dominance” may also be part of the algae development strategies, there are some other unique characteristics of algae that require attention. Unicellular algae have a big surface area in connection with the environment, and also multi-cellular algae cells have a strong exposure to the environment, in contrast to cells in land plants. Despite this strong exposure to the environment algae are able to control environmental parameters around the cells. The internodal cells of *Chara corallina*, for example, can build up several alkaline bands separated by acid bands along the longitudinal axis to facilitate the uptake of inorganic carbon [68,69,70]. This band-formation ability is quite strong and shows that the cells can manipulate the extracellular micro-environment. Due to this acid-alkaline banding pattern, application of the classical “chemiosmotic mechanisms” for auxin transport should only be applied with great care (Figure 1), as pH balance conditions may vary strongly and abruptly along the cell surface length.

## 5. Conclusions

Genetic research and development of molecular biological tools, such as genetic transformation, as well as tools for studying the developmental biology for *Chara* and *Nitella* species is still in its infancy. However, based on our detailed knowledge of auxin function, signaling and transport in land plants, combined molecular and cell physiological research in *Chara* and *Nitella* may open a new page on our understanding of auxin’s central role in plant growth and development.
